# Rethinking the country-level percentage of population residing in urban area with a global harmonized urban definition

**DOI:** 10.1016/j.isci.2024.110125

**Published:** 2024-05-27

**Authors:** Wenyue Li, Yecheng Zhang, Mengxing Li, Ying Long

**Affiliations:** 1 School of Architecture, Tsinghua University, Beijing 100084, China; 2 School of Architecture, Harbin Institute of Technology, Shenzhen 518055, China; 3 Faculty of Information Technology, Monash University, Melbourne, VIC 3800, Australia; 4 Hang Lung Center for Real Estate, Key Laboratory of Ecological Planning & Green Building, Ministry of Education, Tsinghua University, Beijing 100084, China

**Keywords:** urban planning, Human Geography

## Abstract

The UN (United Nations) collects global data on the country-level Percentage of Population Residing in Urban Area (PPRUA). However, variations in urban definitions make these data incomparable across countries. This study assesses national defined PPRUA within UN statistics against estimates we derived using global comparable definitions. Refer to the UN’s Degree of Urbanization framework, we propose 90 global harmonized methods for estimating PPRUA by combining different configurations of three global population datasets, six urban total population thresholds, and five urban population density thresholds. This approach demonstrated significant variations in country-level PPRUA estimations, with wide 95% confidence intervals using the *Z* score method. Most national defined PPRUA fall between the upper 95% CI and the median of the estimations, underscoring the need for globally harmonious PPRUA estimates. This study advocates for a reassessment of datasets and thresholds in the future and for investigating urbanization on a scale beyond the country level.

## Highlights

• Global PPRUA is exhaustively computed using the UN’s proposed methodology
• Estimation configurations result in the inequality of PPRUA outcomes
• The discrepancy between the UN’s PPRUA and our estimates is exposed fully
• Harmonized PPRUA is essential for sustainable urban communities

## Graphical abstract

**Figure:**
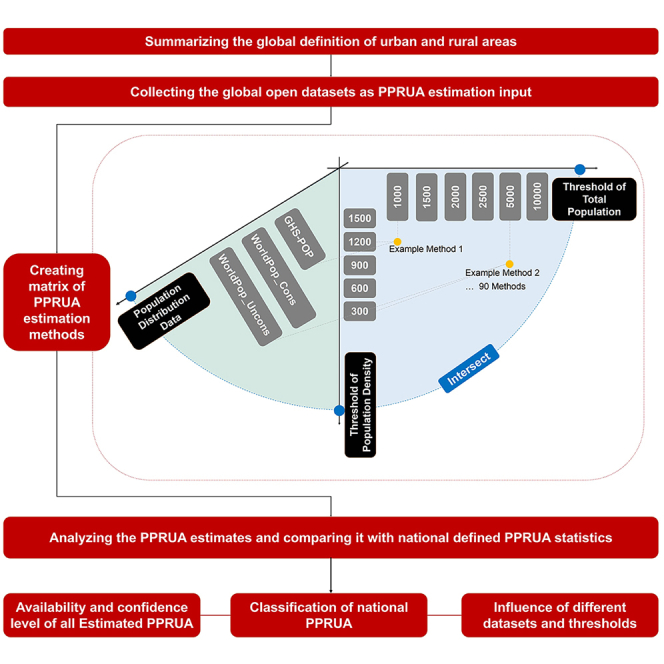

## Introduction

Urbanization is a critical component of economic, societal, and environmental development.^1^^,^^2^^,^^3^ Currently, global urbanization, as a broad socioeconomic phenomenon, not only encompasses the migration of populations from rural to urban areas, but also includes the growth of rural villages through natural population increases until they meet the criteria for being classified as towns. Additionally, it involves the expansion of cities, which grow larger in scope through births, even in the absence of migration, until they absorb their rural surroundings into the urban fabric.^4^ Urbanization is also related to income inequality,^5^ environmental pollution,^6^^,^^7^ energy use,^8^ and emissions.^9^ Properly managed urbanization can play a pivotal role in harnessing the benefits of agglomeration economies and in mitigating environmental degradation and other negative consequences stemming from an increasing urban population.^10^ Accordingly, the rate of urbanization serves as a crucial indicator for policymaking and scientific inquiry.

However, the lack of a standardized global harmonized urban definition complicates the interpretation of urban trends across countries, affecting the accuracy of analyses and posing significant challenges to comparative scientific studies and policy formulations aimed at mitigating urban-related issues such as climate change.^11^ Some researchers have also conducted relevant empirical studies,^12^^,^^13^ utilizing remote sensing data to analyze urban growth in local areas such as East Asia, emphasizing the significant discrepancies in understanding urban expansion due to the lack of a unified urban definition. This inconsistency poses significant challenges to scientists striving for precision in comparative studies. In policy contexts, accurate and comparable urban data are foundational for the success of international agreements, such as the Paris Agreement, which relies on precise urban emission data to guide global climate action. Furthermore, a standardized urbanization metric is instrumental for advancing the Sustainable Development Goals (SDGs), particularly those related to sustainable cities and communities, by providing a common framework for measuring progress. Therefore, adopting a universally accepted definition of urbanization is imperative not only for enhancing research accuracy, but also for the effective development and implementation of policies aimed at tackling global challenges, such as climate change and urban sustainability.^14^

Although globally comparable rate of urbanization is vital for effective development planning, numerous studies depend on country-specific urbanization indicators from the United Nations (UN), which frequently suffer from a lack of comparability. Since the 1990s, the World Urbanization Prospects has reported the global urbanization rate for the UN.^15^ In UN statistics, the urbanization rate is precisely expressed as the PPRUA, based on a range of criteria employed by national governments to differentiate urban from rural areas, with these criteria exhibiting considerable variation across countries. Countries primarily define urban areas using administrative criteria, economic factors, population size/density, or urban characteristics, and many countries have combined these criteria. According to the UN, 121 countries (51.9%) use administrative criteria together with additional criteria to define urban areas: population size/density (108 countries, 16.3%), urban characteristics (69 countries, 29.6%), and economic criteria (38 countries, 16.3%).^16^ In addition, 59 countries (25.3%) use administrative criteria as single definitions of urban areas, which constitutes the most commonly used type of single criterion, ranked by population size/density (37 countries, 15.9%) and urban characteristics (8 countries, 3.4%), while not using single economic criteria. Therefore, the UN’s acceptance of the various definitions supplied by countries for World Urbanization Prospects, based on different designations of urban area, results in the incomparability of PPRUA values.^17^ However, the UN’s statistics remain the primary source for scientists and policy makers worldwide and are employed by entities such as the World Bank.^18^ As global urbanization continues to accelerate, the trajectory of sustainable development is increasingly dependent on the skillful management of urban growth. Misjudging the urbanization rate could thus lead to misguided policies with extensive implications.

The UN, in collaboration with numerous international organizations, has adopted the Degree of Urbanization as a globally unified framework for PPRUA estimation, significantly enhancing the comparability of PPRUA data across countries. The Degree of Urbanization uses 1 km^2^ grids of population density cells to define three types of cells: 1) cities are defined as settlements of at least 50,000 inhabitants in a high-density cluster of grid cells (greater than 1,500 inhabitants per sq. km); 2) towns and semi-dense areas are defined as urban clusters outside cities with at least 5,000 inhabitants in contiguous moderate-density grid cells (at least 300 inhabitants per sq. km); and 3) rural areas are defined as grid cells with a density of less than 300 inhabitants/km^2^, or higher density cells that do not belong to a city. The urban and rural cells are classified using two thresholds: minimum population size and minimum population density. Unlike FUA, the Degree of Urbanization excludes commuting time criteria, enhancing data availability in countries with limited statistics. In recent years, discussions of globally comparable PPRUA have garnered increasing attention from scientists.^2^^,^^18^^,^^19^ Indeed, for measuring PPRUA across countries through a comparable method, a more fundamental issue is the definition of comparable urban areas. For more than a decade, major international organizations have engaged in debates regarding urban definitions in their official documents, with a consensus forming around the Degree of Urbanization as a universal standard. In 2009, the World Bank adopted a harmonized method for urban definitions, as detailed by Buys^15^ and Nelson.^20^ The World Bank’s method considers population size, density, and a certain travel time by road to define urban areas, based on the exogeneous points of urban settlements, which is not exactly harmonized. In 2012, the OECD introduced the Functional Urban Area (FUA) definition, similar to that of the World Bank.^21^ The OECD definition involves three steps: defining core municipalities using gridded population data, connecting non-contiguous cores belonging to the same functional area, and identifying urban hinterlands. The FUA contains both urban cores and hinterlands in space, whose structure is similar to the urban area defined by the World Bank, but it does not use the exogenous points. The FUA can be seen as a global harmonized urban definition, and it has also been endorsed by the UN Statistical Commission as part of the Degree of Urbanization method. In 2020, following thorough consultations, the UN and other entities endorsed the Degree of Urbanization for international statistical comparison,^16^^,^^22^ emphasizing that it complements, not replaces, national PPRUA statistics.

While an international consensus has emerged over the years on adopting the Degree of Urbanization as a globally harmonized method for calculating PPRUA, numerous datasets and thresholds exist for this estimation. However, comprehensive analyses to discern the most robust datasets and thresholds for these estimates are insufficient. In the recently released World Cities Report,^23^ GHS-POP population distribution data were employed to estimate PPRUA. Concurrently, with updates to the national census and advances in remote sensing and computer technology, efforts to decompose national-level population datasets into spatially distributed grids have intensified,^24^^,^^25^ and global population distribution datasets are becoming increasingly abundant. The PPRUA estimates from different datasets vary significantly,^26^ and the outcomes of utilizing other datasets warrant further exploration. Furthermore, the United Nations provided a set of population density and size thresholds to define cities, towns, semi-dense areas, and rural areas, but without sufficient reasons for choosing the thresholds. Scholars have commented that the selection of thresholds appears arbitrary to some extent, despite their transparency and temporal stability over time.^26^ Indeed, the thresholds for the Degree of Urbanization used by the UN are not universal for most countries in defining urban areas. Specifically, the thresholds of cities using the population size threshold of 50,000 from Japan and the population density threshold of 1,500 inhabitants per km^2^ from China are too high for other countries because Japan and China are East Asian countries with the densest population. More critically, while recent studies have presented population shares according to the Degree of Urbanization at the regional level, country-level estimates and analyses for the large-scale testing of different population data and thresholds are still insufficient.^26^^,^^27^^,^^28^

Given that universally comparable country-level PPRUA data are crucial for both policy formulation and scientific research, the prompt development of harmonized datasets is imperative. In World City Report 2022, the Degree of Urbanization approach proposes two levels of understanding with distinct classes of human settlement by analyzing grid cells of one square kilometer, one of them classifying the cells into three classes of area, and the other into six. Whatever the categories, the Degree of Urbanization defines different urbanized areas through population agglomeration. In this article, when referring to Degree of Urbanization, we define urban areas as areas with a higher degree of population agglomeration, which are settlements of a certain scale by interconnected high population density grid cells. The economic and lifestyle criteria are not included in the definition of urban, for they are harder to harmonize globally. What’s more, as the Degree of Urbanization framework emphasizes that the urban and rural are continuous instead of binary in space, using a series of numbers under all reasonable sets of thresholds and datasets can more sufficiently describe the PPRUA. In this study, referring to the framework of the degree of urbanization, we estimated the PPRUA at the country level using multiple reasonable datasets and thresholds. Using population grid data with a spatial resolution of 1 km^2^, we classified the cell grids according to population density thresholds and identified the adjacent cell groups that met the conditions through the total population thresholds, thus determining the scope of the city. To obtain a robust estimation method for the PPRUA, we comprehensively reviewed global open population data and selected a threshold range that meets both common national scenarios and equal intervals. In this regard, we assessed the comparability of the PPRUA by national definition and discussed the datasets and threshold choices for the Degree of Urbanization.

## Results

### The rational datasets and thresholds for the estimation of PPRUA

To identify the most appropriate datasets for the global harmonized PPRUA estimation, we searched all possible global population open datasets to find datasets of global population distributions with a spatial resolution of 1 km^2^ or 30 arc-seconds and constant updates since 2020. The following are the main global population datasets (cf. Table 1): 1) GHS-POP datasets from the European Commission Joint Research Center (Freire et al., 2016); 2) WorldPop datasets from the University of Southampton.^29^ WorldPop uses constrained (WorldPop_Cons) and unconstrained (WorldPop_Uncons) decomposition methods to form two datasets; 3) LandScan datasets from the Oak Ridge National Laboratory^30^; and 4) GPWv4 datasets from the Center for International Earth Science Information Network (CIESIN)^31^ and Columbia University.^32^ Specifically, GPWv4 uses a spatial averaging method based on administrative boundaries with demographic statistics and tends to be average, which means that it's not suitable for globally harmonized country-level PPRUA estimation. LandScan does not measure the residential population, but the average of day- and night-time populations and is thus unsuitable.^26^ GHS-POP and WorldPop have been applied to the Degree of Urbanization in recent studies.^26^^,^^27^ Both use population data collected by CIESIN as input, but adopt different methods to disaggregate the population. The GHS-POP dataset uses the GHS-BUILT dataset of built-up areas to disaggregate the population in the census unit,^33^ which is more in line with the shape of built-up areas as more disaggregated than GPWv4. WorldPop data were divided into WorldPop Constrained global mosaic (WorldPop_Cons) and WorldPop Unconstrained global mosaic (WorldPop_Uncons) data. WorldPop_Uncons distributes population estimates across all land grid cells, while the WorldPop_Cons confines population estimates to identified built-up areas only. We thus identified three rational datasets (GHS-POP, WorldPop_Cons, and WorldPop_Uncons) that yield stable PPRUA estimates.

**Table 1:** Global population distribution datasets

No.	Name	Time span	Resolution	Sources	Author(s)
1	Global Human Settlement Layer-Population (GHS-POP)	1975-2020 at 5-year intervals	250 m/1 km/9 arc-seconds/30 arc-seconds	European Commission Joint Research Center (JRC) and CIESIN, Columbia University	(Freire et al., 2016)^33^
2	WorldPop Constrained Global Mosaic (WorldPop_Cons)	2020	3 arc-seconds	University of Southampton	(Stevens et al., 2015; Tatem et al., 2017)^29^^,^^34^
3	WorldPop Unconstrained Global Mosaic (WorldPop_Uncons)	2000–2020 at 5-year intervals	30 arc-seconds		
4	LandScan Global Population Database (LandScan)	2000–2021	30 arc-seconds	Oak Ridge National Laboratory (ORNL)	(Dobson, 2000)^30^
5	Gridded Population of World (GPW)	2000–2020 at 5-year intervals	30 arc-seconds	Center for International Earth Science Information Network (CIESIN), Columbia University	(Doxsey-Whitfeld, 2015)^32^

Generation process of WorldPop Constrained Global Mosaic: the Constrained Individual countries 2020 downloaded by WorldPop official website(https://hub.worldpop.org/project/categories?id=3) are merged into a global mosaic, and then the resolution is resampled from 3 arc-seconds to 30 arc-seconds.

As many of them have multiple time points, we harmonized the selected data in time using the latest data from 2020 as the input. Furthermore, to address the gap in globally harmonized PPRUA data regarding country-level estimations, country-level administrative boundaries are needed to create country-level zonal statistics of the total and urban population. We thus adopted country-level administrative boundaries from the Global Administrative Unit Layers (GAUL) provided by the Food and Agriculture Organization of the United Nations.^35^

**Figure 1:**
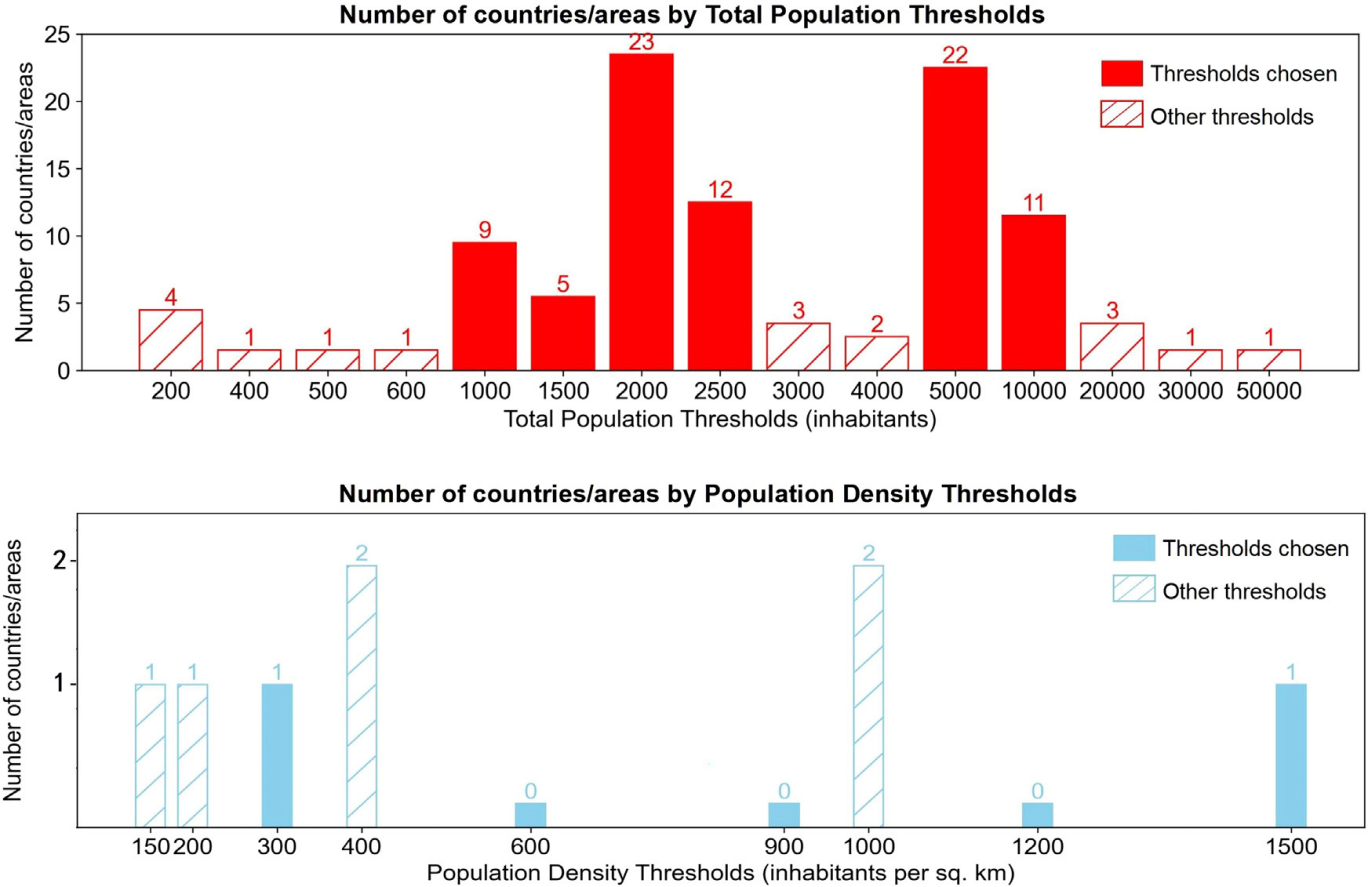
Thresholds of population size and population density in urban definition, from the UN The solid bar charts represent the thresholds selected as rational ones and used in the estimation configurations in this article.

For the thresholds of total population and population density for PPRUA estimation, we counted the number of countries with the above thresholds in the definition of urbanized areas according to the UN World Urbanization Prospect (cf. Figure 1) to select the most commonly used rational thresholds. From Figure 1, we can see that the thresholds of population size of more than 2000 inhabitants are used in 23 countries/areas worldwide, which is the most popular threshold for the total population, followed by 5000, 2500, 10000, 1000, and 1500 inhabitants. We selected all of the above six thresholds of the total population as rational for defining urban areas, and they were all used in at least five countries/areas. The threshold of population density does not appear as common as the thresholds of the total population, with only two countries/areas, respectively, using 400 and 1000 inhabitants per sq. km as thresholds to define urban areas, which are the most frequently utilized and thus considered rational in this study. As there are three kinds of population density thresholds lower than 400 inhabitants per square kilometer that only appeared once, we selected a range with regular intervals above 300 inhabitants per square kilometer and included the thresholds used for the Degree of Urbanization. In total, we selected five population density thresholds and six total population thresholds as rational thresholds to estimate PPRUA at the country level. Compared with the UN’s PPRUA estimation with only one set of datasets and thresholds, our research describes the PPRUA more comprehensively.

To sufficiently list and analyze reasonable numbers of globally harmonized PPRUA estimations at the country level, we proposed 90 configurations of PPRUA estimation methods (the combinations of different population distribution datasets, population density thresholds, and total population thresholds) by integrating the three population distribution datasets, six thresholds for population size, and five thresholds for population density. Globally, we estimated the PPRUA for the entire planet using these 90 configurations of PPRUA estimation methods, yielding results with a 95% confidence interval (CI) for the global PPRUA estimation, ranging from 45.3% to 86.8%. The UN’s official 2018 estimate of the global PPRUA (55.3%) is within this range.

### The PPRUA estimations at the country level

At the country level, we estimated the PPRUA for each country or area using 90 configurations within the Degree of Urbanization. The analysis revealed that a majority of countries/areas yielded valid PPRUA estimates across all 90 configurations. In total, the Global Administrative Unit Layers (GAUL) have 221 spatial units of countries/areas, while there are 233 countries/areas in the statistics of the United Nations. This study presents PPRUA estimates for the 221 countries/areas delineated by GAUL. However, some countries/areas among the 221 countries still have invalid estimations because their territorial areas are so small that they cannot include any population or urban population grid units, such as Monaco, Kosovo, Liechtenstein, Maldives, and many other island countries. Table 2 enumerates the countries/areas with invalid total population and urbanization population statistics for all 90 PPRUA estimation configurations of the Degree of Urbanization; valid PPRUA estimation requires both the total population and urbanization population to be valid. The numbers of countries/areas with invalid total and urban population data differ across the datasets, among which the GHS-POP datasets have the fewest countries/areas with invalid urban population estimation (cf. Table 2), while the Worldpop_Uncons and Worldpop_Cons datasets have the most. These results suggest that, in terms of comprehensive data coverage, the Worldpop_Cons datasets may be less suitable for PPRUA estimation.

**Table 2:** Validity of Estimation results about countries' total population and urban population by different configurations

Dataset	Valid results of total population	Valid results of urban population	The countries with invalid total/urban population estimation																	
WorldPop_Cons	194	188	**ABY**	**AKC**	**APS**	**CIV**	**PRK**	**FLK**	FRO	**GUF**	**ATF**	GRL	**GLP**	**HLT**	**HMD**	**IMT**	**JKM**	**KIL**	KIR	LSO
			**MTQ**	**MTS**	**MYT**	**ANT**	**NIU**	PLW	**REU**	**SPM**	**SGS**	**SJM**	**SWZ**	**TWN**	VIR	**WBG**	**ESH**			
WorldPop_Uncons	214	191	ABY	AKC	**ATF**	**CIV**	DMA	FLK	FRO	FSM	GRD	GRL	HLT	**HMD**	IMT	ISL	KIL	KIR	**KMR**	MNE
			MTS	NIU	PLW	**PSE**	**SGS**	SJM	SPM	SSD	**SWZ**	TON	VIR	WSM						
GHS-POP	207	198	ABY	AKC	**ATF**	**CIV**	FLK	FRO	FSM	GRL	HMD	**IMT**	**JKM**	**KIL**	**KIR**	**KMR**	**MTS**	PLW	**PSE**	**REU**
			**SGS**	**SJM**	**SPM**	**SWZ**	VIR													

Validity means that the country has effective PPRUA estimation in a specific scenario, and invalidity means that the national territorial areas are so small that cannot include any population or urban population grid unit.

All countries or regions names and abbreviations in the Global Administrative Unit Layers (GAUL) can be found in the supplemental information (see Table S1).

The countries with their abbreviation in bold font lack both valid estimations of the countries' urban population and total population. Countries’s abbreviation without bold fonts lack valid estimations of the countries' urban population.

The specific list of invalid estimation results under the selection of different thresholds of population size and population density on countries' urban population can be found in the supplemental information (see Tables S2–S4).

**Figure 2:**
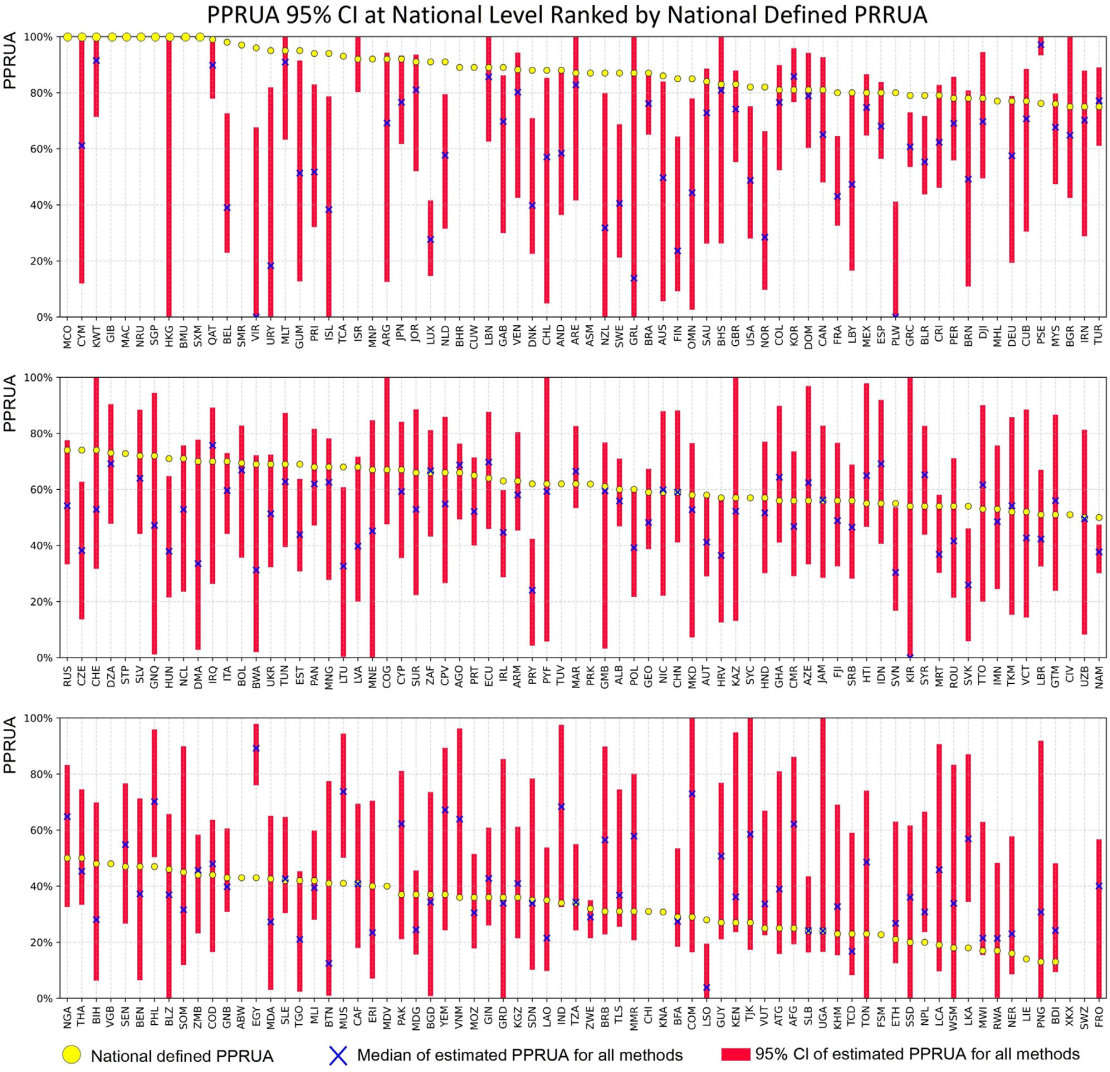
Estimated PPRUA for all scenarios represented by the 95% CI and median at the country level, ranked by national defined PRRUA

For countries/areas with valid PPRUA estimates, we calculated their PPRUA using all 90 configurations (see Tables S6–S8). The estimation results are presented in Figure 2, depicted as median values and 95% confidence intervals (CI) and ranked by the UN’s PPRUA statistics provided by countries. The 95% CI was primarily estimated using the Standard Error of the Mean (SEM), where the SEM is derived from the sample standard deviation divided by the square root of the sample size. To calculate the 95% CI, we employed the formula: Mean±(1.96 ∗ SEM), where 1.96 is the *Z* score associated with a 95% CI under the assumption of a normal distribution. The PPRUA statistics, based on national definitions from UN data, are also included in Figure 2 to facilitate comparison. Figure 2 illustrates that the country-level PPRUA estimations exhibit a wide 95% CI range, indicating that different configurations lead to very different PPRUA estimates. At the same time, as the PPRUA in the UN statistics for different countries and regions are also based on different datasets and methods, the wide range of the 95% CI also suggests that the PPRUA in the UN statistics might have substantial inter-country variability by national definition. We also tested the average and standard deviation of the 95% CI in countries with different income categories and found that income categories did not affect the interval width.

### The classification of national defined PPRUA in UN statistics

**Figure 3:**
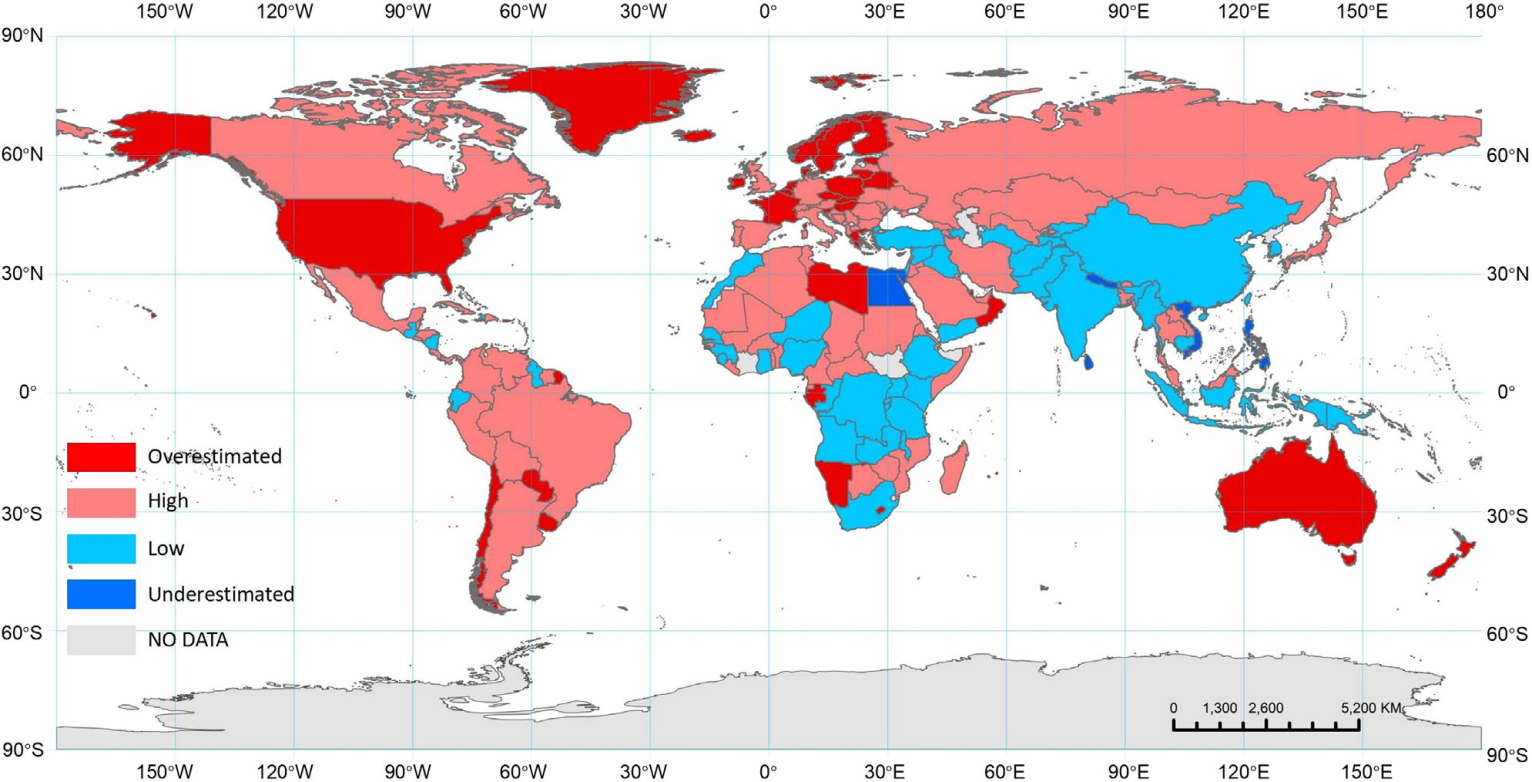
Classification of national PPRUA (1) “Overestimated”: UN’s PPRUA > upper limit of estimated PPRUA 95% CI. 2) “High”: Median of estimated PPRUA 95% CI < UN’s PPRUA ≤ upper limit of estimated PPRUA 95% CI. 3) “Low”: lower limit of estimated PPRUA 95% CI ≤ UN’s PPRUA < median of estimated PPRUA 95% CI. 4) “Underestimated”: UN’s PPRUA < lower limit of estimated PPRUA 95% CI. 5) Because of the lack of UN statistics or the unavailability of PPRUA estimation results (cf. Table 2), NO DATA means there is no data for these countries to consider their classification (see Table S5). This map does not represent the official views or positions of any country or organization, nor does it imply the recognition or denial of any sovereignty or territorial claims. This map is for reference and learning purposes only and has no legal effect.

Owing to varying estimation criteria, the UN’s PPRUA statistics are not directly comparable between countries. Utilizing the global PPRUA estimates from the 90 configurations of the Degree of Urbanization, we categorized national PPRUA in the UN statistics into four categories: “Overestimated,” “High,” “Low,” and “Underestimated.” (see Table S5). The classification standard can be found in the STAR Methods section. The results show that 35 countries (17.4%) have an “Overestimated” national defined PPRUA statistic compared with the estimates, and 90 (44.8%) have “High,” 67 (33.3%) “Low,” and 9 (4.5%) “Underestimated” PPRUA statistics. Because of the lack of UN statistics or the unavailability of PPRUA estimation results (cf. Table 2) for some countries or areas, 201 countries in total are included in the classification (cf. Figure 3).

**Figure 4:**
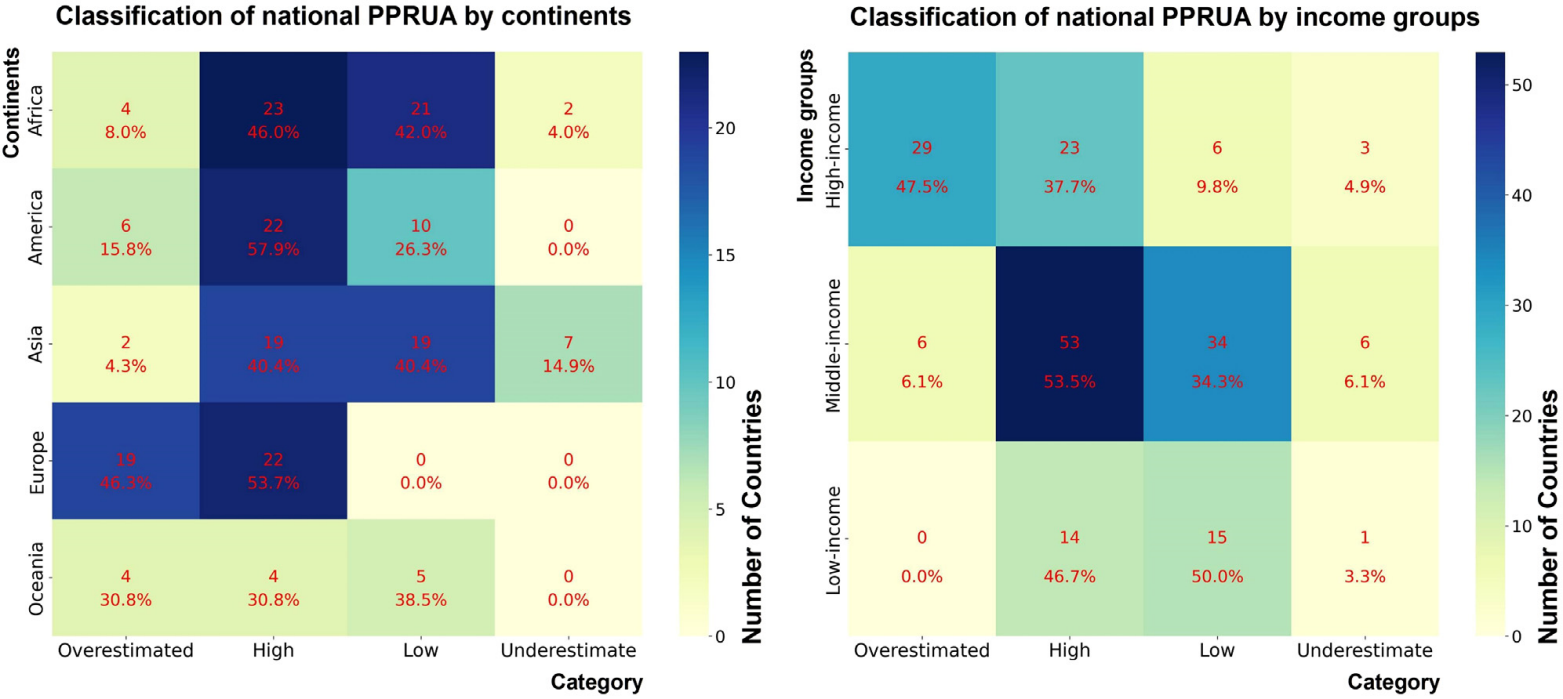
Classification of national PPRUA by continents and income groups The upper number in each thermal grid represents the number of countries, and the lower percentage represents the proportion of the number of countries in the continent or income category in the deviation category to the total number of countries in the category.

Our analysis revealed distinct regional patterns in the classification of national defined PPRUA. For each category, we further quantified the number and proportion of countries across different continents to delineate their agglomeration characteristics (cf. Figure 4A). European countries only exhibit “Overestimated” (19 countries, 46.3%) and “High” (22 countries, 53.7%) national defined PPRUA statistics. African and Asian countries mostly show “High” (23 African countries, 46.0%; 19 Asian countries, 40.4%) and “Low” (21 Africa countries, 42.0%; 19 Asian countries, 40.4%) PPRUA statistics. Oceania and American countries are distributed in only the “Overestimated,” “High,” and “Low” categories, with Oceania countries are more evenly distributed and American countries mostly falling in the “High” (22 countries, 57.9%) category. The above distribution regularities of the categories in the continents may be related to the development level of the respective continent; therefore, we classified countries according to their income based on the UN classification and further analyzed the classification results of the PPRUA in each country.

Our analysis revealed a significant correlation between a country’s income group and its national defined PPRUA category. First, countries in the high-income group are predominantly classified as “Overestimated” (29 countries, 47.5%) and “High” (23 countries, 37.7%). Second, of middle-income countries, 53.5% (53 countries) and 34.3% (34 countries) fall in the “High” and “Low” categories, respectively, and the rest are evenly distributed in the “Overestimated” (6 countries, 6.1%) and “Underestimated” (6 countries, 6.1%) categories. Third, low income-countries are almost entirely concentrated in the “High” (14 countries, 46.7%) and “Low” (15 countries, 50.0%) categories. The predominance of “Overestimated” PPRUA in high-income countries may stem from their national criteria, which often regard broader areas with small and low-density settlements as urbanized, yet the majority of our estimation configurations may not sufficiently encompass such areas.

To verify the rationality of the results, we focused on the eight main countries with the highest GDP as examples, comparing the definition of urban population from the national statistics and the estimation configurations, and examining the factors contributing to the estimation results. years (Table 3). Among the eight countries, the estimated PPRUA 95% CI ranges of the United States in North America and France in Western Europe are higher than the national defined PPRUA statistic while those of the other countries all include the national defined PPRUA statistics. Regarding the definition of urban areas, 7 of the 10 countries considered the population of settlements, and among them, the United States and France defined urban as settlements with populations of more than 2,500 and 2,000, respectively, which constitute smaller population thresholds and broader urban definitions. Other countries with urban definitions that consider settlement populations include Japan, the United Kingdom, India, and Italy. The minimum settlement population size in their national urban definition ranges from 5,000 to 50,000, with larger population thresholds and stricter urban definitions than the former countries. As the United States and France have lower population thresholds for urban settlements, the national defined PPRUA statistics of these countries are higher. Furthermore, among the eight countries, China and India are two developing Asian countries with high population densities, and they have lower national PPRUA statistics than the median estimated PPRUA. This is because these two countries have comprehensive urban definitions, in addition to population density thresholds, and some poorly developed high-density rural areas in these two countries are classified as urban according to the estimation method. Overall, although the urban definition of the above countries includes more than just the population, the above finding can still confirm the rationality of the PPRUA estimation to some degree.

**Table 3:** Definition of Urban Population for the top ten countries with the highest GDP

GDP Rank	Country,Sub-region	Definition (latest)	National PPRUA/Classification of national PPRUA	PPRUA 95% CI range by estimation
1	The United States, North America	Densely settled territory that meets minimum population density requirements and with 2,500 inhabitants or more. A change in the definition for the 2000 census from place-based to density-based affects the comparability of estimates before and after this date.	82.3%^a^ /Overestimated	75.1%–27.8%
2	China, Eastern Asia	Population of city districts with average population density of at least 1,500 persons per square kilometre, population of suburban-district units and township-level units meeting certain criteria, such as having contiguous built-up area, being the location of the local government, or being a street (jiedao) or having a resident committee. Residents living in villages or towns in outer urban and suburban areas that are directly connected to municipal infrastructure and that receive public services from urban municipalities.	59.2% /Low	88.1%–41.1%
3	Japan, Eastern Asia	Cities defined as shi. In general, shi refers to a municipality that satisfies the following conditions: (1) 50,000 inhabitants or more; (2) 60 per cent or more of the houses located in the main built-up areas; (3) 60 per cent or more of the population (including their dependents) engaged in manufacturing, trade or other urban type of business.	91.6% /High	93.1%–61.7%
4	Germany, Western Europe	Communes (kreisfreie Staedte and Kreise) with at least 150 inhabitants per square kilometre.	77.3% /High	78.7%–19.3%
5	The United Kingdom, Northern Europe	Settlements with 10,000 inhabitants or more. For 1971 and earlier, administrative boundaries were used.	83.4% /High	87.9%–55.3%
6	India, Southern Asia	Statutory places with a municipality, corporation, cantonment board or notified town area committee and places satisfying all of the following three criteria: (1) 5,000 inhabitants or more; (2) at least 75 per cent of male working population engaged in non-agricultural pursuits; and (3) at least 400 inhabitants per square kilometre.	34.0% /Low	97.5%–32.5%
7	France, Western Europe	Based on the concept of urban unit, namely communes with 2,000 inhabitants or more in dwellings separated by at most 200 m.	80.4%^a^ /Overestimated	64.5%–32.6%
8	Italy, Southern Europe	Communes with 10,000 inhabitants or more.	70.4% /High	73.0%–44.1%

a means the UN’s statistic of the country is outside the 95% CI of estimation results.

### The influence of different datasets and thresholds on PPRUA estimations by degree of urbanization

**Figure 5:**
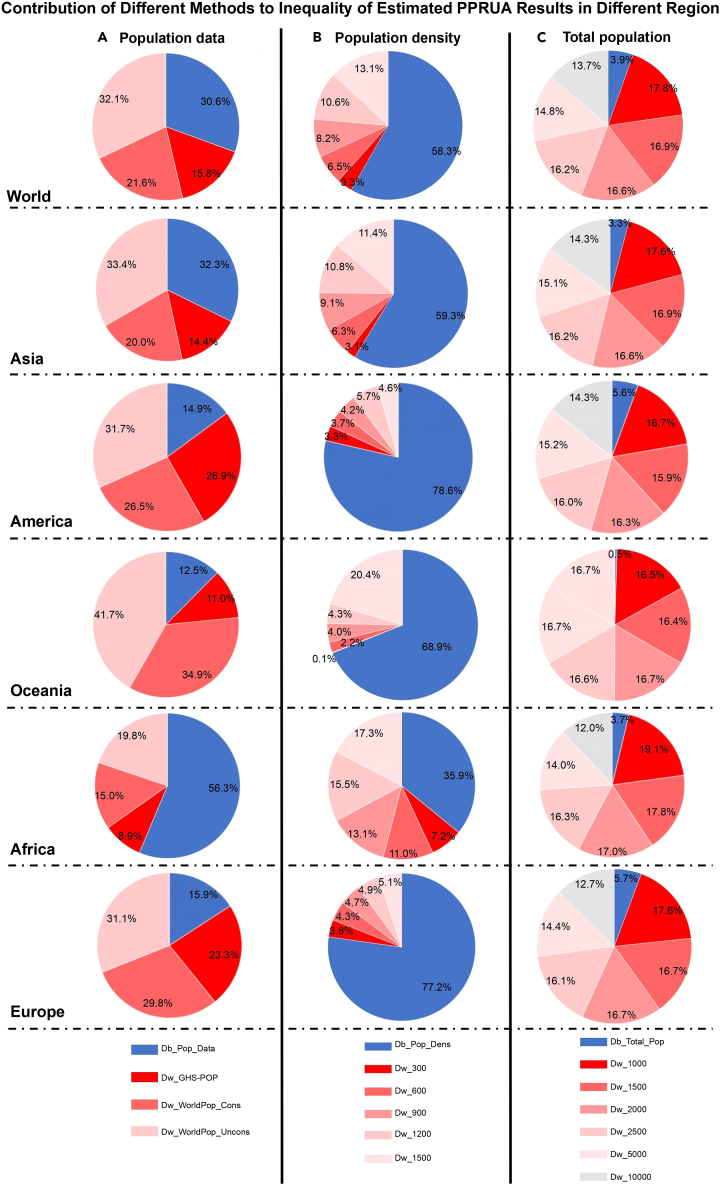
Contribution of different data and thresholds to the inequality of estimated PPRUA results in different regions Blue sector represents the proportion of between group inequality (Disparity across categories. A higher Db indicates more divergence between groups), and red sector represents the proportion of the within group inequality for each subgroup (Disparity within a category. A higher Dw points to more disparity within that specific group). (A) Inequality of the results decomposed by different population distribution data; (B) Inequality of the results decomposed by different population density threshold; (C) Inequality of the results decomposed by total population threshold.

Given the substantial variation in PPRUA estimations under different configurations, it is essential to comprehend the impact of diverse thresholds and datasets to interpret these estimates accurately. To evaluate the contribution of different datasets and thresholds to the inequality of the PPRUA estimates, we grouped the global PPRUA estimates in different configurations by different population distribution datasets (cf. Figure 5A), different thresholds of urban population density (cf. Figure 5B), and different thresholds of urban total population (cf. Figure 5C). Thereafter, we calculated the proportion of between- and within-group inequality to the total inequality of the PPRUA estimations using the decomposed Theil entropy index. In Figure 5, Db is the proportion of between-group inequality, where a higher number indicates greater inequality between the groups, and Dw is the proportion of within-group inequality for each subgroup, where the higher the number, the greater the inequality inside the subgroups.

For between-group inequality, by comparing the Db (cf. Figure 5) percentile for World, it is evident that the differences between groups divided by population distribution datasets and thresholds of population density account for 30.6% and 58.3%, respectively, which are obviously higher than that for groups divided by thresholds of the total population (17.8%). Thus, the difference in datasets and population density thresholds contribute more to the difference in PPRUA estimations than the total population thresholds. For within-group inequality, we analyzed the Dw for World using different grouping configurations, in the following. From Figure 5A, according to the number of Dw indicators, the difference within PPRUA estimations using GHS-POP and WorldPop_cons datasets contribute smaller to the total inequality of the 90 estimations, that is to say PPRUA estimations using Worldpop_Uncons datasets are most different between each other. Given that the UN utilized the GHS-POP dataset in the World Cities Report 2022, our findings shed light on the fact that the use of the GHS-POP dataset reduced the PPRUA estimation differences caused by different thresholds. From Figure 5B, it is evident that the smaller the thresholds of population density, the smaller the PPRUA estimation inequality within the groups. As shown in Figure 5C, the inequality of the PPRUA estimations within groups divided by a larger number of total population thresholds is similar. The abovementioned findings imply that it is important to compare PPRUA estimations with different thresholds of population density, and that higher thresholds can increase the estimation inequality caused by different population distribution datasets and thresholds of the total population. Considering that the UN’s World Cities Report 2022 defines cities using 1500 inhabitants per sq. km as the threshold of population density by degree of urbanization, we believe that from the perspective of PPRUA estimation stability, the adoption of such a high threshold potentially precipitates substantial variability in estimations across diverse population distribution datasets.

## Discussion

In this article, the PPRUA results obtained by using 90 configurations in the framework of the degree of urbanization are not consistent with the existing UN data, thereby significantly revealing the incomparability of UN statistics between countries. We carefully selected three datasets of global population distribution, six thresholds of total population, and five thresholds of population density as rational datasets and thresholds for the global country-level PPRUA estimations based on the degree of urbanization methodology. By crossing the datasets and thresholds presented above, we obtained 90 globally comparable estimation configurations to estimate the PPRUA at the country level. Furthermore, based on the estimations, the classification of national defined PPRUA was evaluated. Our findings reveal not only the different 95% CI and median of country-level PPRUA by global comparable methods across different configuration parameters but also their comparison with the UN’s statistics. These differences are particularly influenced by the nation’s economic development, which highlights the challenges of accurately estimating a globally unified PPRUA using current methodologies. These findings provide pivotal insights into sustainable development and the assessment of urban environments globally.

Through our analysis, several implications emerge for both researchers and policy-making entities. Our results indicate that varying PPRUA estimation configurations yield significantly divergent results. Additionally, PPRUA statistics with incomparability, as defined nationally, are unsuitable for global comparative analysis. Hence, we recommend that researchers thoroughly consider and analyze the inherent incomparability of national defined PPRUA before using such data. For organizations, we strongly advocate the urgent development and application of country-level PPRUA estimations grounded in a globally harmonized urban definition. Specifically, we find that different population distribution datasets and different population density thresholds contribute significantly to the inequality of the different estimations by the 90 configurations. Based on the results of the global comparable estimation, we classified the PPRUA estimation by national definition into four categories, and we find that the European countries and high-income countries are mainly with “Overestimated” and “High” PPRUA statistics by national definition.

The findings of this article emphasize that estimating a globally harmonized PPRUA necessitates a meticulous selection of datasets and thresholds, a task that poses its own evaluation challenges. First, when it comes to thresholds, we strongly resonate with the approach in the UN’s World Cities Report 2022,^23^ which employs multiple distinct sets of thresholds to delineate areas with varying urban characteristics, as urban and rural areas are continuous rather than binary. Second, for the production of global population distribution datasets, the approaches of population data allocation can be divided into two methods. The spatial averaging method decomposes the census data spatially into census units,^36^^,^^34^^,^^37^ and the spatial modeling method appropriately improves the spatial accuracy by adding auxiliary geographic datasets.^38^ An implementation of the spatial averaging method is seen in GPWv4, which uses administrative boundaries to predict population distributions. Building upon this, GHS-POP takes this a step further by basing its estimates on the population data provided by GPWv4, but it refines these estimates using mapped built-up areas (GHS-BUILT), as noted by Freire.^33^ This process disaggregates the population more finely within census units, with its distribution being more influenced by the physical shape and layout of buildings than by GPWv4 alone. WorldPop, on the other hand, employs a random forest algorithm to allocate population across space. It produces two versions: the WorldPop_Cons, which confines population estimates to build-up areas identified via detailed mapping, and the WorldPop_Uncons, which distributes population estimates to all land grid cells, potentially leading to misallocations in uninhabited areas and underestimation of urban populations. The varied methodologies of GHS-POP and WorldPop result in significant differences in the accuracy of their outcomes, particularly regarding the alignment of population distributions with actual human settlements. Given these nuances, GHS-POP reflects building shapes and locations more accurately for its refined approach to population estimation. In addition, we also found the use of the GHS-POP reduced the PPRUA estimation differences caused by different thresholds. Thus, we recommend the use of GHS-POP as a superior alternative for globally harmonized country-level PPRUA estimations.

The significance of calculating the globally comparable PPRUA and its difference from national statistics does not imply that all countries must abandon their current definitions in favor of an internationally comparable one, as countries at different levels of development have different objectives for urban definitions. More importantly, this implies that some studies using UN’s published statistical data as a source of predictive indicators need to be aware of data incomparability. The themes of such studies include, but are not limited to, urbanization and its correlation with carbon emissions, pollution, urban heat islands, and levels of urban infrastructure. The results of this study will facilitate the acquisition of globally comparable urbanization rate data (estimates of PPRUA for all countries under all scenarios can be found in Tables S6, S7, and S8) for more accurate research and more solid evidence-based policy decisions. Taking carbon emissions as an example, on the one hand, numerous empirical studies on the correlation between urbanization and carbon emissions in different regions and periods based on national statistical data have reached varied conclusions,^39^^,^^40^^,^^41^ such as positive, negative, bidirectional, nonlinear, and insignificant correlations, where inconsistency in statistical calibers may have led to vague or even incorrect conclusions. On the other hand, some studies have already proven that the impact of urbanization on CO_2_ emissions varies significantly between different countries and regions, thus proposing differentiated urban development policy recommendations for different countries.^42^^,^^43^ For example, there exists an inverted U-shaped relationship between urbanization and carbon emissions, meaning that carbon emissions increase before reaching a certain urbanization threshold (approximately 73.80%), and then decrease afterward. Therefore, in connection to policy recommendations, this study suggests that for developing regions such as Asia, reaching this urbanization threshold means that countries can reduce carbon emissions through efficient urban planning and management. However, in reality, our research finds that the population density threshold for urban definitions in Asian countries, such as China, is much higher than that in European countries. Compared to Europe, the PPRUA statistical data in Asian countries in UN’s statistic tends to underestimate, suggesting that continuing urban expansion, as opposed to focusing more on sustainable development and efficient energy use, may not be the optimal choice for densely populated Asian countries.^44^

### Limitations of the study

By computing a globally unified country-level PPRUA with all the rational datasets and configuration parameters, the findings suggest that relying solely on a single data acquisition method is inadequate for generating globally comparable PPRUA data. This underscores the need for further research to identify more reliable methods of PPRUA calculations. Although this study conducted globally comparable PPRUA estimates at the country level, it did not provide precise figures for each country’s PPRUA, which seems to be a limitation. Nonetheless, the primary contribution of this study is to prompt authorities and scholars to reflect on the complexities of PPRUA estimation. Our discussion and dissemination of authorities are very important to confirm the finally selected method. Owing to limited space, this study only investigated PPRUA estimations at the country level; however, the rationality of urban area identification under different methods has not been thoroughly explored at the city level. Future studies should evaluate the rationality of the datasets and thresholds by comparing the reasonableness of urban space definitions^45^^,^^46^^,^^47^^,^^48^^,^^49^^,^^50^ under different PPRUA estimation configurations.^51^

## STAR★Methods

### Key resources table

**Table:** 

REAGENT or RESOURCE	SOURCE	IDENTIFIER
**Deposited data**		

Global human settlement layer-population	Columbia University	https://ghsl.jrc.ec.europa.eu/index.php
WorldPop constrained global mosaic	University of Southampton	https://hub.worldpop.org/
WorldPop unconstrained global mosaic	University of Southampton	https://hub.worldpop.org/
LandScan global population database	Oak Ridge National Laboratory	https://landscan.ornl.gov/
Gridded population of world	Columbia University	https://sedac.ciesin.columbia.edu/data/collection/gpw-v4/sets/browse
Global administrative unit layers	Food and Agriculture Organization of the United Nations	https://www.eea.europa.eu/en/datahub/datahubitem-view/de85b91a-5594-4be9-91ba-feea218d32d7

**Software and algorithms**		

ArcGIS 10.5	Environmental Systems Research Institute	ArcGIS for Desktop Basic (RRID: SCR_011081)
SPSS 22	International Business Machines Corporation	IBM SPSS Statistics (RRID: SCR_016479)

### Resource availability

#### Lead contact

Further information and requests for resources should be directed to and will be fulfilled by the lead contact, Ying Long (ylong@tsinghua.edu.cn).

#### Materials availability

This study did not generate new unique materials.

#### Data and code availability

The data used in this study are all available from public data resources that have been appropriately cited in the manuscript. Code and any other additional information required to re-analyse the data reported in this paper is available from the lead contact upon request.

### Method details

#### Summarize the appropriate thresholds and datasets

First, we summarize the definitions of urban and rural areas in each country worldwide and select the rational thresholds of not only the total population but also the population density. Second, we collected rational global open datasets of population distribution as inputs to support PPRUA estimation. By reviewing the generation process and data characteristics of the global population datasets, we determined the appropriate population data combination to be GHS-POP, WorldPop_Cons, and WorldPop_Uncons by comparing their stability.

#### Estimating the country level PPRUA by 90 configurations

In this article, a matrix comprising appropriate datasets and commonly used thresholds was used to estimate globally comparable PPRUA ranges. The matrix defines the urban area using the thresholds of the total population and population density based on population distribution datasets, referencing the framework of the Degree of Urbanization. Different combinations of datasets and thresholds were designed using different calculation configurations. Specifically, the matrix includes three axes: population distribution data, population density threshold, and total population threshold. The 6 total population thresholds and 5 population density thresholds from the 2 axes can be paired to generate 30 combinations, and then coordinating the combinations with the 3 population distribution datasets can create 90 estimation configurations.

Based on the abovementioned methods, we use the ArcMap 10.2 software to estimate the country-level PPRUA. According to the population distribution data, areas with eligible population densities are delimited as urban areas for the calculation of the urban population, and the national administrative boundary is used for the calculation of the national population. Then, by using zonal statistics, we can ascertain the urban and national populations of each country, and the ratio of the two was the estimated value of the PPRUA. In the calculation above, we assume the size of each population distribution grid to be 1 square kilometer, and the resolution of the population distribution datasets we use are approximately 1 km/30 arc-second.

#### Classifying the national defined PPRUA

As the different PPRUA estimation configurations make the UN’s statistics incomparable between countries, referencing the PPRUA estimates, we classified the UN’s national PPRUA statistic into differentcategories, to help us compare national PPRUA statistics comprehensively. We compared the UN’s national PPRUA statistic with our estimated PPRUA, and divided the comparison results of different countries into the following four categories. 1) Overestimated: UN’s national PPRUA number higher than the higher value of the estimated PPRUA 95% CI. 2) High: UN PPRUA is lower than the higher value of the estimated PPRUA 95% CI and higher than the median of the estimated PPRUA 95% CI. 3) Low: UN PPRUA is lower than the median of the estimated PPRUA 95% CI. 4) Underestimated: UN PPRUA is lower than the lower value of the estimated PPRUA 95% CI.

#### Detecting the sensitivity of the estimations

The Theil index is used to detect the sensitivity of the estimated PPRUA by measuring the influence of different datasets and thresholds on the inequality of PPRUA estimations. To make the results simpler and more intuitive, the Theil index is calculated using data at the global level instead of the country level. The advantage of the Theil index is that it can be decomposed as follows: When the sample is divided into multiple groups, the Thiel index can measure the contribution of the between- and within-group inequality to the total inequality. The Theil index (T) in our study is defined as:

(Equation 1) $$T=\frac{1}{n}{\sum_{i=1}^{n}\frac{x_{i}}{\bar{x}}\ln\left(\frac{x_{i}}{\bar{x}}\right)=T}_{B}+T_{W}=\sum_{k=1}^{K}y_{k}\;\ln\;\left(\frac{y_{k}}{n_{k}/n}\right)+\sum_{k=1}^{K}y_{k}\left(\sum_{i\in g_{k}}\frac{y_{i}}{y_{k}}\ln\frac{y_{i}/y_{k}}{1/n_{k}}\right)$$

where $x_{i}$ is the number of the PPRUA estimations for the whole world, and $\bar{x}$ is the average number of all the 90 estimations. $T_{B}$ and $T_{W}$ represent the Theil index between and within groups, and their sum is equal to the overall Theil index. $g_{k}$ is the name of the group, K is the number of groups, k is the rank of the group (k=1,2,…,K), n is the total number of the estimations (90), $n_{k}$ is the number of estimations in $g_{k}$, $y_{i}$ is the proportion of $x_{i}$ to the sum of all estimates, and $y_{k}$ is the proportions of all the $x_{i}$ in $g_{k}$ to the sum of all the estimates. In this paper, we grouped the data based on different population distribution data, different thresholds of population density, and different thresholds of the total population, and the number of the corresponding groups are 4, 3 and 6. Db_group and $D_{W}\_g_{k}$ respectively represent the contribution ratio of between group inequality and within group inequality for each group to the total inequality. The proportion of between group inequality, and the proportion of within group inequality for all groups is 100%.

(Equation 2) $$Db\_group=\frac{T_{b}}{T}=\frac{\sum_{k=1}^{K}y_{k}\;\ln\;\left(\frac{y_{k}}{n_{k}/n}\right)}{T}$$

(Equation 3) $$Dw\_g_{k}=\frac{T_{w}}{T}=\frac{y_{k}\sum_{i\in g_{k}}\frac{y_{i}}{y_{k}}\ln\;\left(\frac{y_{i}/y_{k}}{1/n_{k}}\right)}{T}$$
