# Supplementary material for: Rethinking the country-level percentage of population residing in urban area with a global harmonized urban definition
Source: iScience. 2024 May 27;27(6):110125. doi: 10.1016/j.isci.2024.110125 (PMC11186970; doi:10.1016/j.isci.2024.110125)
Supplement: Document S1. Tables S1–S5 [file mmc1.pdf]

iScience, Volume *27*

Supplemental information

Rethinking the country-level percentage of population residing in urban area with a global harmonized urban definition

Wenyue Li, Yecheng Zhang, Mengxing Li, and Ying Long

Supplementary Information

Table S1. The full names of countries involved in this article corresponding to the abbreviations, related to Table 2.

Countries in Global Administrative Unit Layers and existing in the international standard ISO3166[a]							
Full names		Full names		Full names		Full names	
Afghanistan	AFG	Egypt	EGY	Lebanon	LBN	Samoa	WSM
Albania	ALB	El Salvador	SLV	Lesotho	LSO	Sao Tome and Principe	STP
Algeria	DZA	Equatorial Guinea	GNQ	Liberia	LBR	Saudi Arabia	SAU
Andorra	AND	Eritrea	ERI	Libya	LBY	Senegal	SEN
Angola	AGO	Estonia	EST	Lithuania	LTU	Serbia	SRB
Antigua and Barbuda	ATG	Ethiopia	ETH	Luxembourg	LUX	Sierra Leone	SLE
Argentina	ARG	Falkland Islands (Malvinas)	FLK	Madagascar	MDG	Singapore	SGP
Armenia	ARM	Faroe Islands	FRO	Malawi	MWI	Slovakia	SVK
Australia	AUS	Micronesia (Federated States of)	FSM	Malaysia	MYS	Slovenia	SVN
Austria	AUT	Fiji	FJI	Mali	MLI	Solomon Islands	SLB
Azerbaijan	AZE	Finland	FIN	Malta	MLT	Somalia	SOM
Bahamas	BHS	France	FRA	Martinique	MTQ	South Africa	ZAF
Bahrain	BHR	French Guiana	GUF	Mauritania	MRT	South Georgia and the South Sandwich Islands	SGS
Bangladesh	BGD	French Polynesia	PYF	Mauritius	MUS	South Sudan	SSD
Barbados	BRB	French Southern and Antarctic Territories	ATF	Mayotte	MYT	Spain	ESP
Belarus	BLR	Gabon	GAB	Mexico	MEX	Sri Lanka	LKA
Belgium	BEL	Gambia	GMB	Moldova, Republic of	MDA	State of Palestine	PSE
Belize	BLZ	Georgia	GEO	Mongolia	MNG	Sudan	SDN
Benin	BEN	Germany	DEU	Montenegro	MNE	Suriname	SUR
Bhutan	BTN	Ghana	GHA	Morocco	MAR	Svalbard and Jan Mayen Islands	SJM
Bolivia	BOL	Greece	GRC	Mozambique	MOZ	Swaziland	SWZ
Bosnia and Herzegovina	BIH	Greenland	GRL	Myanmar	MMR	Sweden	SWE
Botswana	BWA	Grenada	GRD	Namibia	NAM	Switzerland	CHE
Brazil	BRA	Guadeloupe	GLP	Nepal	NPL	Syrian Arab Republic	SYR

Brunei Darussalam	BRN	Guam	GUM	Netherlands	NLD	Taiwan	TWN
Bulgaria	BGR	Guatemala	GTM	Netherlands Antilles	ANT	Tajikistan	TJK
Burkina Faso	BFA	Guinea	GIN	New Caledonia	NCL	Thailand	THA
Burundi	BDI	Guinea-Bissau	GNB	New Zealand	NZL	The former Yugoslav Republic of Macedonia	MKD
Côte d'Ivoire	CIV	Guyana	GUY	Nicaragua	NIC	Timor-Leste	TLS
Cambodia	KHM	Haiti	HTI	Niger	NER	Togo	TGO
Cameroon	CMR	Heard Island and McDonald Islands	HMD	Nigeria	NGA	Tonga	TON
Canada	CAN	Honduras	HND	Niue	NIU	Trinidad and Tobago	TTO
Cape Verde	CPV	Hong Kong	HKG	Norway	NOR	Tunisia	TUN
Cayman Islands	CYM	Hungary	HUN	Oman	OMN	Turkey	TUR
Central African Republic	CAF	Iceland	ISL	Pakistan	PAK	Turkmenistan	TKM
Chad	TCD	India	IND	Palau	PLW	U.K. of Great Britain and Northern Ireland	GBR
Chile	CHL	Indonesia	IDN	Panama	PAN	Uganda	UGA
China	CHN	Iran (Islamic Republic of)	IRN	Papua New Guinea	PNG	Ukraine	UKR
Colombia	COL	Iraq	IRQ	Paraguay	PRY	United Arab Emirates	ARE
Comoros	COM	Ireland	IRL	Peru	PER	United Republic of Tanzania	TZA
Congo	COG	Isle of Man	IMN	Philippines	PHL	United States of America	USA
Costa Rica	CRI	Israel	ISR	Poland	POL	United States Virgin Islands	VIR
Croatia	HRV	Italy	ITA	Portugal	PRT	Uruguay	URY
Cuba	CUB	Jamaica	JAM	Puerto Rico	PRI	Uzbekistan	UZB
Cyprus	CYP	Japan	JPN	Qatar	QAT	Vanuatu	VUT
Czech Republic	CZE	Jordan	JOR	Republic of Korea	KOR	Venezuela	VEN
Dem Peoples Rep of Korea	PRK	Kazakhstan	KAZ	Réunion	REU	Viet Nam	VNM
Democratic Republic of the Congo	COD	Kenya	KEN	Romania	ROU	West Bank	WBG
Denmark	DNK	Kiribati	KIR	Russian Federation	RUS	Western Sahara	ESH
Djibouti	DJI	Kuwait	KWT	Rwanda	RWA	Yemen	YEM
Dominica	DMA	Kyrgyzstan	KGZ	Saint Lucia	LCA	Zambia	ZMB
Dominican Republic	DOM	Lao People's Democratic	LAO	Saint Pierre et Miquelon	SPM	Zimbabwe	ZWE

		Republic					
Ecuador	ECU	Latvia	LVA	Saint Vincent and the Grenadines	VCT		
Countries in Global Administrative Unit Layers that do not exist in the international standard ISO3166[b]							
Abyei	ABY	Gaza Strip	GZS	Jammu and Kashmir	JKM	Ma'tan al-Sarra	MTS
Aksai Chin	AKC	Hala'ib triangle	HLT	Kingman Reef	KMR		
Arunachal Pradesh	APS	Ilemi triangle	IMT	Kuril islands	KIL		

Table S2. Countries' urban population with existing invalid estimation results by different configurations in WorldPop_Cons, related to Table 2.

Country	300_1000	300_1500	300_2000	300_2500	300_5000	300_10000	600_1000	600_1500	600_2000	600_2500	600_5000	600_10000	900_1000	900_1500	900_2000	900_2500	900_5000	900_10000	1200_1000	1200_1500	1200_2000	1200_2500	1200_5000	1200_10000	1500_1000	1500_1500	1500_2000	1500_2500	1500_5000	1500_10000
ABY	×	×	×	×	×	×	×	×	×	×	×	×	×	×	×	×	×	×	×	×	×	×	×	×	×	×	×	×	×	×
AKC	×	×	×	×	×	×	×	×	×	×	×	×	×	×	×	×	×	×	×	×	×	×	×	×	×	×	×	×	×	×
ATF	×	×	×	×	×	×	×	×	×	×	×	×	×	×	×	×	×	×	×	×	×	×	×	×	×	×	×	×	×	×
CIV	×	×	×	×	×	×	×	×	×	×	×	×	×	×	×	×	×	×	×	×	×	×	×	×	×	×	×	×	×	×
DMA	×	×	×	×	×	×	×	×	×	×	×	×	×	×	×	×	×	×	×	×	×	×	×	×	×	×	×	×	×	×
FLK	×	×	×	×	×	×	×	×	×	×	×	×	×	×	×	×	×	×	×	×	×	×	×	×	×	×	×	×	×	×
FRO	○	○	○	○	○	○	○	○	○	○	○	○	○	○	○	○	○	○	○	○	○	×	×	×	○	×	×	×	×	×
FSM	×	×	×	×	×	×	×	×	×	×	×	×	×	×	×	×	×	×	×	×	×	×	×	×	×	×	×	×	×	×
GRD	×	×	×	×	×	×	×	×	×	×	×	×	×	×	×	×	×	×	×	×	×	×	×	×	×	×	×	×	×	×
GRL	○	○	○	○	○	○	○	○	○	○	○	○	○	○	○	○	○	×	○	○	○	○	×	×	○	○	○	○	×	×
HLT	×	×	×	×	×	×	×	×	×	×	×	×	×	×	×	×	×	×	×	×	×	×	×	×	×	×	×	×	×	×
HMD	×	×	×	×	×	×	×	×	×	×	×	×	×	×	×	×	×	×	×	×	×	×	×	×	×	×	×	×	×	×
IMT	×	×	×	×	×	×	×	×	×	×	×	×	×	×	×	×	×	×	×	×	×	×	×	×	×	×	×	×	×	×
ISL	×	×	×	×	×	×	×	×	×	×	×	×	×	×	×	×	×	×	×	×	×	×	×	×	×	×	×	×	×	×
KIL	×	×	×	×	×	×	×	×	×	×	×	×	×	×	×	×	×	×	×	×	×	×	×	×	×	×	×	×	×	×
KIR	○	○	○	×	×	×	×	×	×	×	×	×	×	×	×	×	×	×	×	×	×	×	×	×	×	×	×	×	×	×
KMR	×	×	×	×	×	×	×	×	×	×	×	×	×	×	×	×	×	×	×	×	×	×	×	×	×	×	×	×	×	×
MNE	×	×	×	×	×	×	×	×	×	×	×	×	×	×	×	×	×	×	×	×	×	×	×	×	×	×	×	×	×	×
MTS	×	×	×	×	×	×	×	×	×	×	×	×	×	×	×	×	×	×	×	×	×	×	×	×	×	×	×	×	×	×
NIU	×	×	×	×	×	×	×	×	×	×	×	×	×	×	×	×	×	×	×	×	×	×	×	×	×	×	×	×	×	×
PLW	○	○	○	○	×	×	○	○	○	○	×	×	○	○	○	○	×	×	○	○	○	○	×	×	○	○	○	○	×	×
PSE	×	×	×	×	×	×	×	×	×	×	×	×	×	×	×	×	×	×	×	×	×	×	×	×	×	×	×	×	×	×
SGS	×	×	×	×	×	×	×	×	×	×	×	×	×	×	×	×	×	×	×	×	×	×	×	×	×	×	×	×	×	×
SJM	×	×	×	×	×	×	×	×	×	×	×	×	×	×	×	×	×	×	×	×	×	×	×	×	×	×	×	×	×	×
SPM	×	×	×	×	×	×	×	×	×	×	×	×	×	×	×	×	×	×	×	×	×	×	×	×	×	×	×	×	×	×
SSD	×	×	×	×	×	×	×	×	×	×	×	×	×	×	×	×	×	×	×	×	×	×	×	×	×	×	×	×	×	×
SWZ	×	×	×	×	×	×	×	×	×	×	×	×	×	×	×	×	×	×	×	×	×	×	×	×	×	×	×	×	×	×

TON	×	×	×	×	×	×	×	×	×	×	×	×	×	×	×	×	×	×	×	×	×	×	×	×	×	×	×	×	×	×
VIR	○	○	○	○	○	○	○	○	○	○	○	×	○	○	○	○	×	×	○	○	×	×	×	×	×	×	×	×	×	×
WSM	×	×	×	×	×	×	×	×	×	×	×	×	×	×	×	×	×	×	×	×	×	×	×	×	×	×	×	×	×	×

Table S3. Countries' urban population with existing invalid estimation results by different configurations in WorldPop_Uncons, related to Table 2.

Country	300_1000	300_1500	300_2000	300_2500	300_5000	300_10000	600_1000	600_1500	600_2000	600_2500	600_5000	600_10000	900_1000	900_1500	900_2000	900_2500	900_5000	900_10000	1200_1000	1200_1500	1200_2000	1200_2500	1200_5000	1200_10000	1500_1000	1500_1500	1500_2000	1500_2500	1500_5000	1500_10000
ABY	×	×	×	×	×	×	×	×	×	×	×	×	×	×	×	×	×	×	×	×	×	×	×	×	×	×	×	×	×	×
AKC	×	×	×	×	×	×	×	×	×	×	×	×	×	×	×	×	×	×	×	×	×	×	×	×	×	×	×	×	×	×
ATF	×	×	×	×	×	×	×	×	×	×	×	×	×	×	×	×	×	×	×	×	×	×	×	×	×	×	×	×	×	×
CIV	×	×	×	×	×	×	×	×	×	×	×	×	×	×	×	×	×	×	×	×	×	×	×	×	×	×	×	×	×	×
DMA	○	○	○	○	○	○	○	○	○	○	○	○	○	○	○	○	○	○	○	○	○	○	○	○	○	○	○	○	○	×
FLK	×	×	×	×	×	×	×	×	×	×	×	×	×	×	×	×	×	×	×	×	×	×	×	×	×	×	×	×	×	×
FRO	○	○	○	○	○	○	○	○	○	○	○	○	○	×	×	×	×	×	×	×	×	×	×	×	×	×	×	×	×	×
FSM	○	○	○	○	○	○	○	○	○	○	○	×	○	○	○	○	○	○	×	○	○	○	○	×	○	○	○	○	○	×
GRD	○	○	○	○	○	○	○	○	○	○	○	○	○	○	○	○	○	○	○	○	○	○	○	○	○	○	○	○	○	×
GRL	○	○	○	○	○	○	×	×	×	×	×	×	×	×	×	×	×	×	×	×	×	×	×	×	×	×	×	×	×	×
HLT	×	×	×	×	×	×	×	×	×	×	×	×	×	×	×	×	×	×	×	×	×	×	×	×	×	×	×	×	×	×
HMD	×	×	×	×	×	×	×	×	×	×	×	×	×	×	×	×	×	×	×	×	×	×	×	×	×	×	×	×	×	×
IMT	×	×	×	×	×	×	×	×	×	×	×	×	×	×	×	×	×	×	×	×	×	×	×	×	×	×	×	×	×	×
ISL	○	○	○	○	○	○	○	○	○	○	○	○	○	○	○	○	○	○	○	○	○	○	○	○	○	×	×	×	×	×
KIL	○	×	×	×	×	×	×	×	×	×	×	×	×	×	×	×	×	×	×	×	×	×	×	×	×	×	×	×	×	×
KIR	○	○	○	×	×	×	○	○	×	×	×	×	×	×	×	×	×	×	×	×	×	×	×	×	×	×	×	×	×	×
KMR	×	×	×	×	×	×	×	×	×	×	×	×	×	×	×	×	×	×	×	×	×	×	×	×	×	×	×	×	×	×
MNE	○	○	○	○	○	○	○	○	○	○	○	○	○	○	○	○	○	○	○	○	○	○	○	○	×	×	×	×	×	×
MTS	×	×	×	×	×	×	×	×	×	×	×	×	×	×	×	×	×	×	×	×	×	×	×	×	×	×	×	×	×	×
NIU	×	×	×	×	×	×	×	×	×	×	×	×	×	×	×	×	×	×	×	×	×	×	×	×	×	×	×	×	×	×
PLW	○	○	○	○	×	×	×	×	×	×	×	×	×	×	×	×	×	×	×	×	×	×	×	×	×	×	×	×	×	×
PSE	×	×	×	×	×	×	×	×	×	×	×	×	×	×	×	×	×	×	×	×	×	×	×	×	×	×	×	×	×	×
SGS	×	×	×	×	×	×	×	×	×	×	×	×	×	×	×	×	×	×	×	×	×	×	×	×	×	×	×	×	×	×
SJM	×	×	×	×	×	×	×	×	×	×	×	×	×	×	×	×	×	×	×	×	×	×	×	×	×	×	×	×	×	×
SPM	×	×	×	×	×	×	×	×	×	×	×	×	×	×	×	×	×	×	×	×	×	×	×	×	×	×	×	×	×	×
SSD	○	○	○	○	○	○	○	○	○	○	○	○	○	○	○	○	○	○	○	○	○	○	○	○	○	○	○	○	×	×
SWZ	×	×	×	×	×	×	×	×	×	×	×	×	×	×	×	×	×	×	×	×	×	×	×	×	×	×	×	×	×	×
TON	○	○	○	○	○	○	○	○	○	○	○	○	○	○	○	○	○	○	○	○	○	○	○	○	○	×	×	×	×	×
VIR	○	○	○	○	○	○	○	○	○	○	○	×	×	×	×	×	×	×	×	×	×	×	×	×	×	×	×	×	×	×
WSM	○	○	○	○	○	○	○	○	○	○	○	○	○	○	○	○	○	○	○	○	○	○	×	×	×	×	×	×	×	×

Table S4. Countries' urban population with existing invalid estimation results by

different configurations in GHS-POP, related to Table 2.

Country	300_1000	300_1500	300_2000	300_2500	300_5000	300_10000	600_1000	600_1500	600_2000	600_2500	600_5000	600_10000	900_1000	900_1500	900_2000	900_2500	900_5000	900_10000	1200_1000	1200_1500	1200_2000	1200_2500	1200_5000	1200_10000	1500_1000	1500_1500	1500_2000	1500_2500	1500_5000	1500_10000
ABY	○	○	○	○	○	○	○	○	○	○	○	○	○	○	○	○	○	○	○	○	○	○	○	○	○	○	○	○	○	×
AKC	○	○	○	○	×	×	○	○	○	○	×	×	○	○	○	○	×	×	○	○	○	○	×	×	○	○	○	○	×	×
ATF	×	×	×	×	×	×	×	×	×	×	×	×	×	×	×	×	×	×	×	×	×	×	×	×	×	×	×	×	×	×
CIV	×	×	×	×	×	×	×	×	×	×	×	×	×	×	×	×	×	×	×	×	×	×	×	×	×	×	×	×	×	×
FLK	○	○	○	×	×	×	×	×	×	×	×	×	×	×	×	×	×	×	×	×	×	×	×	×	×	×	×	×	×	×
FRO	○	○	○	○	○	○	○	○	○	○	○	○	○	○	○	○	○	×	○	○	×	×	×	×	×	×	×	×	×	×
FSM	○	○	○	○	○	○	○	○	○	○	○	○	○	○	○	○	○	○	○	○	○	○	○	○	○	○	○	○	○	×
GRL	○	○	○	○	○	○	○	○	○	○	○	○	○	○	○	○	○	○	○	○	○	○	○	○	○	○	○	×	×	×
HMD	○	○	○	×	×	×	○	○	×	×	×	×	○	○	×	×	×	×	○	○	×	×	×	×	○	×	×	×	×	×
IMT	×	×	×	×	×	×	×	×	×	×	×	×	×	×	×	×	×	×	×	×	×	×	×	×	×	×	×	×	×	×
JKM	×	×	×	×	×	×	×	×	×	×	×	×	×	×	×	×	×	×	×	×	×	×	×	×	×	×	×	×	×	×
KIL	×	×	×	×	×	×	×	×	×	×	×	×	×	×	×	×	×	×	×	×	×	×	×	×	×	×	×	×	×	×
KIR	×	×	×	×	×	×	×	×	×	×	×	×	×	×	×	×	×	×	×	×	×	×	×	×	×	×	×	×	×	×
KMR	×	×	×	×	×	×	×	×	×	×	×	×	×	×	×	×	×	×	×	×	×	×	×	×	×	×	×	×	×	×
MTS	×	×	×	×	×	×	×	×	×	×	×	×	×	×	×	×	×	×	×	×	×	×	×	×	×	×	×	×	×	×
PLW	○	○	○	○	×	×	○	○	○	×	×	×	○	○	×	×	×	×	○	○	×	×	×	×	×	×	×	×	×	×
PSE	×	×	×	×	×	×	×	×	×	×	×	×	×	×	×	×	×	×	×	×	×	×	×	×	×	×	×	×	×	×
REU	×	×	×	×	×	×	×	×	×	×	×	×	×	×	×	×	×	×	×	×	×	×	×	×	×	×	×	×	×	×
SGS	×	×	×	×	×	×	×	×	×	×	×	×	×	×	×	×	×	×	×	×	×	×	×	×	×	×	×	×	×	×
SJM	×	×	×	×	×	×	×	×	×	×	×	×	×	×	×	×	×	×	×	×	×	×	×	×	×	×	×	×	×	×
SPM	×	×	×	×	×	×	×	×	×	×	×	×	×	×	×	×	×	×	×	×	×	×	×	×	×	×	×	×	×	×
VIR	○	○	○	○	○	○	○	○	○	○	○	×	○	○	×	×	×	×	○	○	×	×	×	×	○	○	○	○	×	×

Table S5. Country level consistency evaluation categories of UN PPRUA estimation compared by estimated PPRUA, related to Figure 3.

Overestimated	High				Low		Underestimated	NO DATA*
AND	AGO	DZA	LVA	SVK	AFG	NGA	BDI	ABW[b]
AUS	ALB	ERI	MDA	SVN	ARM	PAK	BGD	ASM[b]
BEL	ARE	ESP	MDG	TCD	ATG	PNG	BHR	BMU[b]
BGR	ARG	EST	MEX	THA	BEN	PSE	COM	CHI[b]
BLR	AUT	FJI	MKD	TKM	BRB	PYF	EGY	CUW[b]
BRA	AZE	GAB	MLI	TUN	CHN	ROU	LKA	CIV[b]
CAN	BFA	GBR	MNE	TUR	COD	SDN	MUS	FRO[a]
CZE	BHS	GEO	MNG	UKR	ECU	SEN	NPL	FSM[b]
DNK	BIH	GHA	MOZ	VEN	ETH	SSD	PHL	GIB[b]
FIN	BLZ	GMB	MRT	ZMB	GIN	SYR	RWA	KNA[b]

FRA	BOL	GNB	MYS	ZWE	GRD	TGO		LIE[b]
GRC	BRN	GNQ	NCL		GTM	TJK		MAC[b]
GRL	BTN	HND	NIC		GUY	TLS		MDV[b]
GUM	BWA	HRV	OMN		HKG	TON		MHL[b]
IRL	CAF	HUN	PAN		HTI	TTO		MNP[b]
ISL	CHE	IRN	PER		IDN	TZA		MCO[b]
KWT	CHL	IRQ	POL		IMN	UGA		NRU[b]
LUX	CMR	ISR	PRK		IND	UZB		SXM[b]
NAM	COG	ITA	PRT		JAM	VCT		SMR[b]
NLD	COL	JOR	PRY		KEN	VNM		STP[b]
NOR	CPV	JPN	RUS		KGZ	VUT		SWZ[a,b]
NZL	CRI	KAZ	SAU		KHM	WSM		SYC[b]
PLW	CUB	KIR	SGP		KOR	YEM		TCA[b]
PRI	CYM	LAO	SLB		LCA	ZAF		TUV[b]
QAT	CYP	LBN	SLE		MAR			VGB[b]
SWE	DEU	LBR	SLV		MLT			XKX[a,b]
URY	DJI	LBY	SOM		MMR			
USA	DMA	LSO	SRB		MWI			
VIR	DOM	LTU	SUR		NER			
